# FIGO best practice guidance for diagnosis and treatment of fibroids

**DOI:** 10.1002/ijgo.70496

**Published:** 2025-08-25

**Authors:** Ivo Meinhold‐Heerlein, Nikhil Purandare, Ally Murji, Paul Fogarty, Jolly Beyeza, Philippe Descamps

**Affiliations:** ^1^ Justus Liebig University Giessen: Justus‐Liebig‐Universitat Giessen Giessen Hesse Germany; ^2^ Galway University Hospital, University of Galway Galway Ireland; ^3^ University of Toronto Toronto Ontario Canada; ^4^ Figo London UK; ^5^ Mulago Specialised Women and Neonatal Hospital Kampala Uganda; ^6^ University Hospital Angers Angers France

**Keywords:** FIGO toolkit, GNRH analogue, leiomyoma, pathogenesis, pregnancy, surgery

When the FIGO Regional Congress 2022 took place in Cartagena, Colombia, several experts came together and decided to improve care for patients with fibroids.

Why?

Because the majority of women worldwide develop fibroids during their reproductive phase. Fibroids can impair bladder, bowel, and sexual function, cause infertility, and lead to complications such as early and late miscarriage, breach presentation, postpartum hemorrhage, and irregular menstrual bleeding with anemia, and may even transform into malignancy.^1^, ^2^, ^3^

Fibroids mainly affect women during their reproductive years, though their effects can persist beyond this period, depending on their size and location. African women bear the highest burden, with an incidence of up to 80%, and they tend to develop larger and more numerous fibroids compared to other populations.^1^

Therefore, it is essential to equip doctors, nurses, and midwives with up‐to‐date knowledge on the pathogenesis, diagnosis, and treatment of fibroids. In regions where the disease burden is high, many health professionals may lack access to the theoretical and practical resources needed to diagnose and manage fibroids effectively.

It was clear from the outset in Cartagena that a free and accessible online resource would benefit health professionals. Since then, members of the four different FIGO committees (Minimal Access Surgery, Menstrual Disorders and Related Health Impacts, Reproductive Medicine, Endocrinology and Infertility, and Childbirth and PPH), representing the five FIGO regions, have joined forces to begin developing this. This online resource is intended to serve as the first FIGO ‘toolkit’ for doctors, healthcare professionals such as nurses and midwives, as well as graduate and postgraduate students. The aim is to remain precise and detailed, yet highly practical.

The first step has been taken with this special issue of FIGO's *International Journal of Gynecology & Obstetrics*, which reviews the current status of fibroid diagnosis and treatment. Senior experts in the field have summarized the existing scientific knowledge and outlined practical treatment approaches, or they have guided younger scientists from the World Association of Trainees in Obstetrics & Gynecology (WATOG) in doing so. We hope this selection of articles engages readers, raises awareness, refreshes knowledge, and shares the latest findings on diagnosing and treating fibroids. In the meantime, we are preparing the next step: the online toolkit. We are currently working on protocols, video clips, and illustrations to provide freely accessible, practical guidance.

We are proud to present the “FIGO Best Practice Guidance for Diagnosis and Treatment of Fibroids,” a cross‐committee initiative launched under the motto “FIGO Fights Fibroids!”

## AUTHOR CONTRIBUTIONS

All authors contributed to the writing and finalizing of the manuscript.

## CONFLICT OF INTEREST STATEMENT

The authors have no conflicts of interest.

## Data Availability

Data sharing is not applicable to this article as no new data were created or analyzed in this study.
